# LiDAR-derived dataset of extracted trees and shrubs from AHN4 airborne laser scanning in the Oostvaardersplassen wetland, the Netherlands

**DOI:** 10.1016/j.dib.2026.113169

**Published:** 2026-08-12

**Authors:** Jinhu Wang, W. Daniel Kissling

**Affiliations:** Institute for Biodiversity and Ecosystem Dynamics (IBED), University of Amsterdam, P.O. Box 94240, 1090 GE Amsterdam, The Netherland

**Keywords:** Reedbeds, Marshes, Voxelization, GeoTIFF, Point clouds, Individualization, Habitat monitoring, Vegetation structure

## Abstract

This article describes a geospatial dataset of trees and shrubs extracted from the fourth Dutch national airborne laser scanning (ALS) survey (AHN4) covering the Oostvaardersplassen wetland in the Netherlands. The dataset was generated in the context of the MAMBO project, which focuses on automated workflows for deriving habitat condition metrics from LiDAR data for European habitat monitoring. The input data consist of nationally acquired AHN4 point clouds (2020–2022). A dedicated processing workflow was applied to classify and extract woody vegetation points in marsh and reedbed environments, where trees and shrubs co-occur with dense herbaceous vegetation.

The workflow includes (i) pre-processing and segmentation of vegetation points, (ii) clustering of tree and shrub points using neighbourhood analysis and voxel-based aggregation, and (iii) delineation of individual woody elements through seed detection and connectivity analysis in three-dimensional space. Accuracy assessments were conducted using manually created ground truth datasets derived from ALS point clouds and by referencing 8 cm resolution aerial imagery for selected validation plots and patches. The dataset includes classified ALS point clouds (LAZ), extracted tree and shrub point clouds (LAZ), derived raster products (GeoTIFF), vector boundary files (ESRI Shapefile), processing scripts, and documentation.

The dataset and associated processing workflow, source code, and metadata are publicly available through Zenodo, GitHub, and the LifeWatch Metadata Catalogue. The dataset can be reused for applications such as habitat structure mapping, woody vegetation monitoring in wetlands, LiDAR-based workflow benchmarking, training and validation of classification algorithms, and upscaling of tree extraction approaches to other airborne laser scanning datasets.

Specifications Table

**Table:** 

Subject	Earth & Environmental Sciences
Specific subject area	Woody vegetation extraction and individualisation from airborne LiDAR for wetland habitat monitoring.
Type of data	Type of data: Point cloud; Raster; Vector; Source code; Metadata; Documentation Data formats: LAZ (raw and classified point clouds); GeoTIFF (raster); ESRI Shapefile (vector); CSV (tabular accuracy metrics); Python scripts (.py); Markdown/Text (README); JSON/XML (metadata) Data processing level: Raw (AHN4 input point clouds); Processed (classified point clouds); Analysed (segmented trees and shrubs, individualized tree clusters); Derived (raster and vector boundary products).
Data collection	Airborne laser scanning data from the fourth national ALS survey of the Netherlands (AHN4, 2020–2022) acquired by the Dutch national mapping programme were used as input. Pre-classified points were processed using a voxel- and neighbourhood-based workflow implemented in C++ and Python to segment woody vegetation and delineate individual trees and shrubs. Parameters (radius, voxel size, minimum points per cluster) were uniformly applied. Accuracy was assessed against manually labelled ground truth from ALS data by referencing 8 cm aerial imagery (https://app.pdok.nl/viewer/#x=160000.00&y=455000.00&z=3.0000&background=BRT-A%20standaard&layers=a301ddc7-c26f-42d8-b367-509ae5ae47d0;Actueel_orthoHR;_;1).
Data source location	The ALS point clouds were obtained from the Dutch national elevation programme AHN (Actueel Hoogtebestand Nederland; https://www.ahn.nl/). The study area is located in the Oostvaardersplassen nature reserve, the Netherlands, with an approximate centre coordinate of 5.418842° E, 52.456870° N. The input data consisted of publicly available AHN4 ALS point clouds accessed via the national data portal (https://www.arcgis.com/home/webscene/viewer.html?layers=77da2e9eeea8427aab2ac83b79097b1a). From the national ALS coverage, only tiles intersecting the boundary of the Oostvaardersplassen were selected. The included tile indices were: 26AN2, 26AZ2, 26BN1, 26BZ1, 20DZ2, 26BN2, and 26BZ2. These LiDAR tiles were subsequently clipped using the boundary polygon (ESRI Shapefile) of the study site to restrict the dataset to the area of interest. The clipped ALS point clouds and the site boundary shapefile are also publicly available via a dedicated repository (https://doi.org/10.48546/workflowhub.datafile.5.1).
Data accessibility	Repository name: Zenodo Data identification number (DOI): 10.5281/zenodo.18511137 (concept DOI); 10.5281/zenodo.18511138 (version 1.0); 10.5281/zenodo.18594751 (version 2); 10.5281/zenodo.19076728 (version 3); 10.5281/zenodo.19704362 (version 4); 10.5281/zenodo.19726739 (version 5); 10.5281/zenodo.20311343 (version 6) Direct URL to data: https://doi.org/10.5281/zenodo.18511137
Related research article	Not available

## 1 Value of the Data

• This dataset [1] provides spatially explicit representations of woody vegetation (trees and shrubs) derived from nationally acquired airborne laser scanning point clouds in a large wetland ecosystem. It includes classified and clipped point clouds (LAZ), extracted woody vegetation points, individualized tree clusters, raster products (GeoTIFF), vector boundaries (ESRI Shapefile), accuracy assessment outputs, and full workflow documentation. The combination of raw input data, processed outputs, and validation material enables transparent inspection of each processing stage.
• The dataset can be reused for the development, benchmarking, and comparison of LiDAR-based vegetation classification and individual tree delineation algorithms [[2], [3], [4]]. Researchers can apply alternative segmentation methods to the same clipped ALS tiles, evaluate parameter sensitivity, or compare performance against the provided ground truth plots and accuracy metrics.
• The openly available workflow and source code enable adaptation of the processing chain to other wetland areas or national ALS datasets. The data structure supports replication of voxel-based clustering, neighbourhood analysis, and connectivity-based tree individualisation approaches [5,6] under comparable environmental conditions.
• The dataset can serve as training and validation material for machine learning applications using three-dimensional point clouds [4,7,8], including supervised classification, object-based analysis, and feature extraction in marsh and reedbed environments.
• The spatial products can be integrated with complementary ecological, hydrological, or habitat datasets to support multi-source analyses of vegetation structure, habitat mapping, and landscape-scale environmental monitoring workflows.

## 2 Background

Airborne laser scanning (ALS) provides three-dimensional point clouds that enable structural characterization of vegetation across large spatial extents [[9], [10], [11]]. In wetland ecosystems such as the Oostvaardersplassen (the Netherlands), woody vegetation occurs in mosaic patterns within reedbeds and marshes, where field-based mapping is demanding. Automated extraction of vegetation structure from ALS data enables the generation of spatially explicit habitat information and supports monitoring frameworks such as Essential Biodiversity Variables (EBVs) [12].

Within the EU-funded MAMBO project (Modern Approaches to the Monitoring of BiOdiversity), workflows are being developed to derive habitat condition metrics from LiDAR data for European-scale monitoring [[13], [14], [15]]. A previously published workflow focused on extracting red deer trails from ALS point clouds in the same wetland system [16]. As red deer move from grasslands into reedbeds to forage on shrubs and trees, spatial information on woody vegetation distribution and structure is required.

In addition, the development of tree classification and individualization methods from airborne LiDAR is relevant for applications beyond site-based case studies [11,17,18]. The present dataset [1] documents the processing chain, intermediate products, and validation data for extracting woody vegetation in marsh environments and supports further development and upscaling of such workflows across larger ALS datasets [[11], [12], [13]].

## 3 Data Description

The dataset is publicly available via Zenodo (DOI: https://doi.org/10.5281/zenodo.18511137) and is organized into seven main folders [1]. The structure is designed to separate input data, processed outputs, scripts, and spatial reference layers. The repository also includes a README file describing the folder structure, file naming conventions, coordinate reference system information, and usage instructions. A summary of the main characteristics of the dataset files is also provided in Table 1. Together, the dataset provides a structured and reproducible collection of input point clouds, processed outputs, validation data, spatial reference layers, and post-processing scripts.

**Table 1:** Overview of the main dataset components provided in the Zenodo repository, including file size, format, coordinate reference system (CRS), and key data attributes for the input point clouds, extracted woody vegetation products, spatial boundary layers, and validation datasets.

Data file	File size	Format	CRS	Main attributes / content
Input points	2.3 GB	ASPRS LAS	Amersfoort / RD New (EPSG:28,992)	Pre-classified ALS point clouds (classification ID = 1) representing vegetation in the study area; X, Y, Z coordinates; intensity; return information
Extracted trees and shrub points	64.2 MB	ASPRS LAS	Amersfoort / RD New (EPSG:28,992)	Segmented woody vegetation points representing extracted trees and shrubs
GeoTIFFs of extracted trees and shrubs	276 MB	GeoTIFF	Amersfoort / RD New (EPSG:28,992)	Rasterized woody vegetation products at 1 m spatial resolution; cell values represent maximum vegetation height
Shapefiles of extracted trees and shrubs	3.88 MB	ESRI Shapefile	Amersfoort / RD New (EPSG:28,992)	Polygon boundaries representing extracted woody vegetation footprints
Shapefiles of marsh area	39.4 KB	ESRI Shapefile	Amersfoort / RD New (EPSG:28,992)	Polygon boundary of the marsh study area used for clipping input ALS data
Shapefiles of nature reserve	10.8 KB	ESRI Shapefile	Amersfoort / RD New (EPSG:28,992)	Polygon boundary of the Oostvaardersplassen nature reserve
Validation data	14.0 MB	ASPRS LAS	Amersfoort / RD New (EPSG:28,992)	Manually annotated validation datasets for woody vegetation classification and tree/shrub individualization

### 3.1 Folder: 1_Input_vegetation_points

**Fig. 1:**
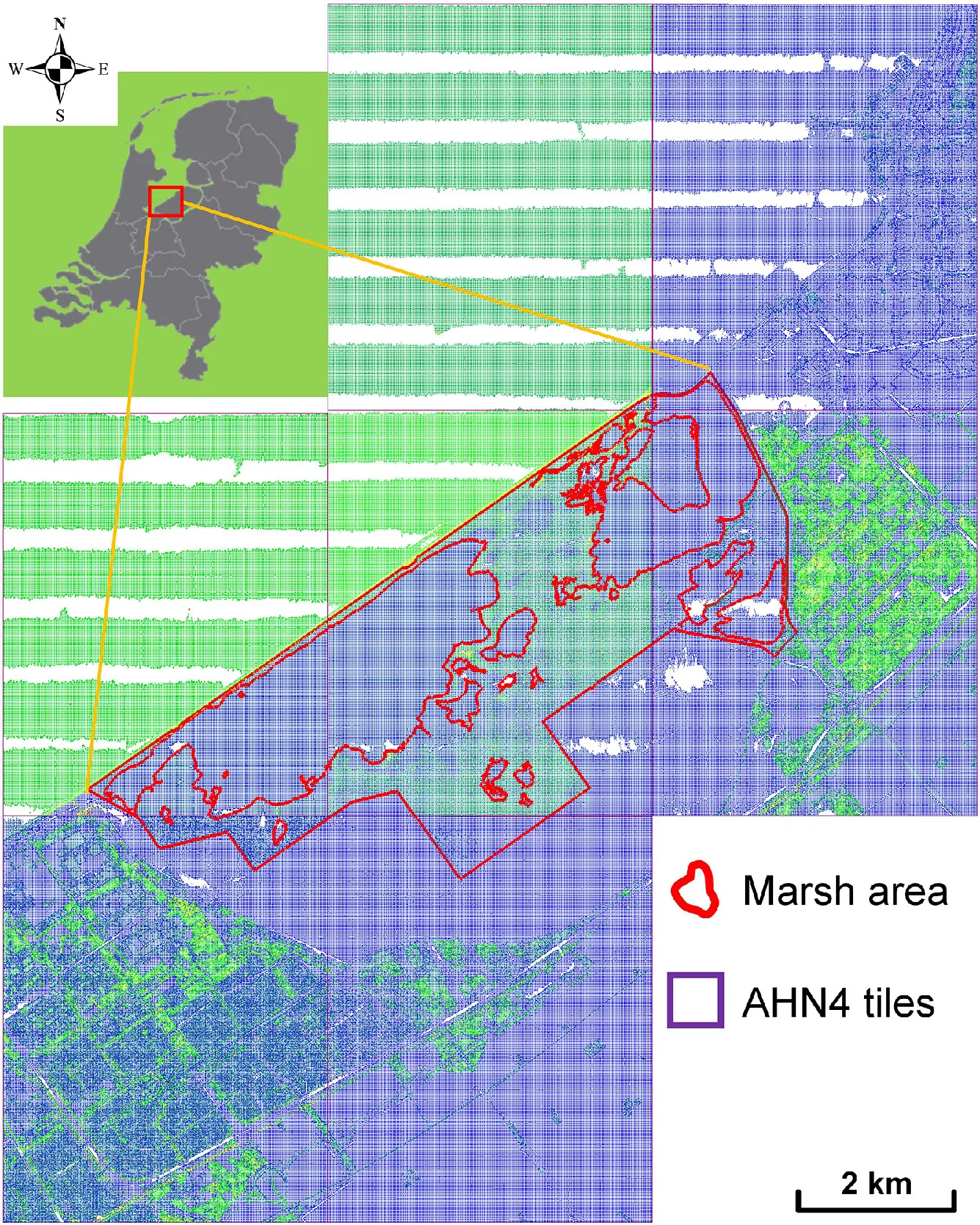
Overview of the marsh area in the Oostvaardersplassen nature reserve (5.418842° E, 52.456870° N), showing the spatial extent of the selected input point cloud tiles from the fourth national airborne laser scanning dataset of the Netherlands (AHN4).

This folder contains the input ALS point clouds used for woody vegetation extraction. The files are subsets of the AHN4 input point cloud tiles, clipped to the marsh area of Oostvaardersplassen (Fig. 1). Only points classified as ``Unclassified'' (ASPRS classification ID = 1) [19] were included. In the AHN4 pre-classification, features such as ground, water, and buildings are assigned dedicated ASPRS classes, whereas vegetation points in the marsh and reedbed environments of the Oostvaardersplassen are predominantly retained as “Unclassified”. These points were therefore used as candidate vegetation points for the woody vegetation extraction workflow. Data are provided in LAZ format.

### 3.2 Folder: 2_Extracted_trees_shrubs_points

**Fig. 2:**
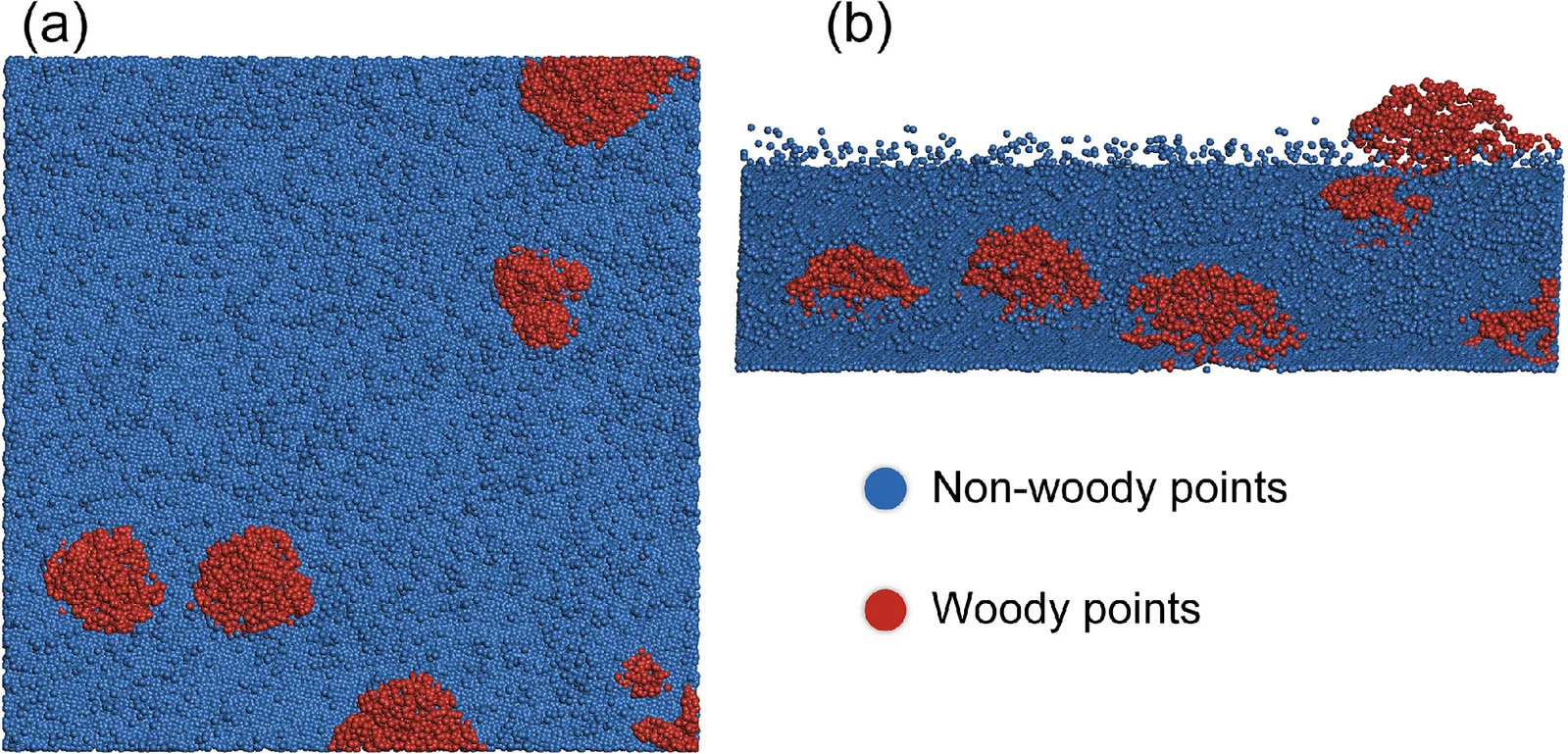
Example visualization of extracted woody vegetation points (in red) within a 50 × 50 m plot, shown in (a) top view and (b) side view.

This folder contains the processed point clouds representing extracted trees and shrubs (Fig. 2). The files are provided in LAZ format and represent the classified woody vegetation points after segmentation and individualization.

### 3.3 Folder: 3_Scripts

This folder contains Python scripts used for post-processing of the extracted woody vegetation point clouds (Table 2). Two scripts are provided:

• 1_generate_tiff.py: Converts extracted LAZ files into raster GeoTIFF products.
• 2_generate_boundary.py: Converts GeoTIFF rasters into vector boundary polygons in ESRI Shapefile format.

**Table 2:** Overview of scripts, input requirements, and output file types.

Script name	Description	Input requirements	Key parameters & operations	Output file type
1_generate_tiff.py	Converts extracted .laz point clouds into raster GeoTIFF Digital Surface Models (DSMs) using a binning algorithm	Folder containing .laz point cloud files	Resolution: 1 m by default. Coordinate reference system (CRS): Hardcoded to EPSG:28,992. Compute: Uses max_workers = os.cpu_count() - 2 for parallel processing.	.tif (GeoTIFF, 32-bit Float)
2_generate_boundary.py	Extracts the valid data footprint from GeoTIFF rasters and converts them into vector boundary polygons	Folder containing .tif raster files	Data masking: Filters out np.nan / 'NoData' values to isolate valid geometries. Geometry: Uses gpd.GeoDataFrame.dissolve() to merge noisy polygons into a single boundary footprint. Compute: Uses max_workers = os.cpu_count() - 2	.shp (ESRI Shapefile)

These scripts enable reproduction of the rasterization and boundary extraction steps.

### 3.4 Folder: 4_GeoTIFFs&shapefiles

**Fig. 3:**
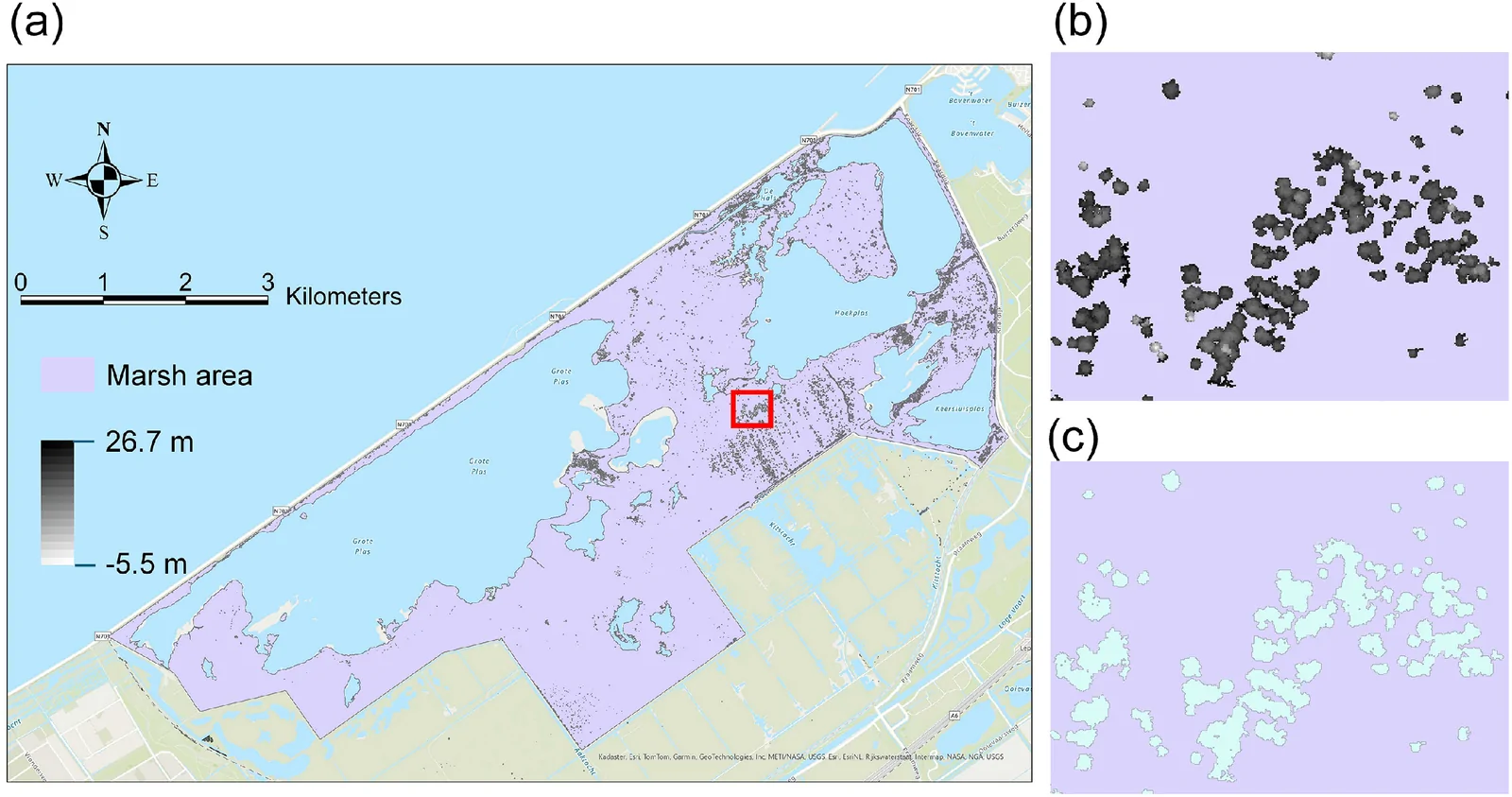
Example of derived raster and vector products representing woody vegetation. (a) Overview of the marsh area in the Oostvaardersplassen showing the GeoTIFF raster of extracted woody vegetation (legends shows elevation relative to sea level). (b) Zoom-in of the raster (red rectangle in a), where cell values (white to black shading) represent the maximum point height within each 1 m grid cell. (c) Corresponding boundary polygons of woody vegetation derived from the raster products.

This folder contains derived spatial products generated from the extracted woody vegetation points covering the whole study area (Fig. 3). Two data formats are provided:

• GeoTIFF rasters at 1 m spatial resolution representing trees and shrubs.
• ESRI Shapefiles representing extracted woody vegetation boundaries derived from the raster products.

These files provide gridded and vector representations of woody vegetation distribution.

### 3.5 Folder: 5_Marsh_area

This folder contains the boundary polygon (ESRI Shapefile) of the marsh area within the Oostvaardersplassen (Fig. 1). This polygon was used to spatially clip and constrain the input vegetation point clouds to the marsh extent.

### 3.6 Folder: 6_OVP_area

This folder contains the boundary polygon (ESRI Shapefile) of the entire Oostvaardersplassen nature reserve. This file defines the broader spatial extent of the study area.

### 3.7 Folder: 7_Accuracy_evaluation_datasets

This folder contains manually annotated datasets used for validating woody vegetation classification and individualization, including 20 square plots (50 m × 50 m) for classification assessment and 20 woody vegetation patches for individualization validation.

## 4 Experimental Design, Materials and Methods

This section documents the full processing chain from national ALS input tiles to segmented and individualized woody vegetation point clouds, rasterized outputs, vector boundaries, and validation datasets.

### 4.1 Input data acquisition

The input data consist of ALS point clouds from the fourth national elevation dataset of the Netherlands (AHN4; acquisition period 2020–2022). The data were acquired within the Dutch national mapping programme using airborne LiDAR systems operated under standardized national specifications. The delivered point clouds are georeferenced in the Dutch national coordinate reference system (EPSG:28,992, Amersfoort / RD New) and provided in LAZ format.

**Fig. 4:**
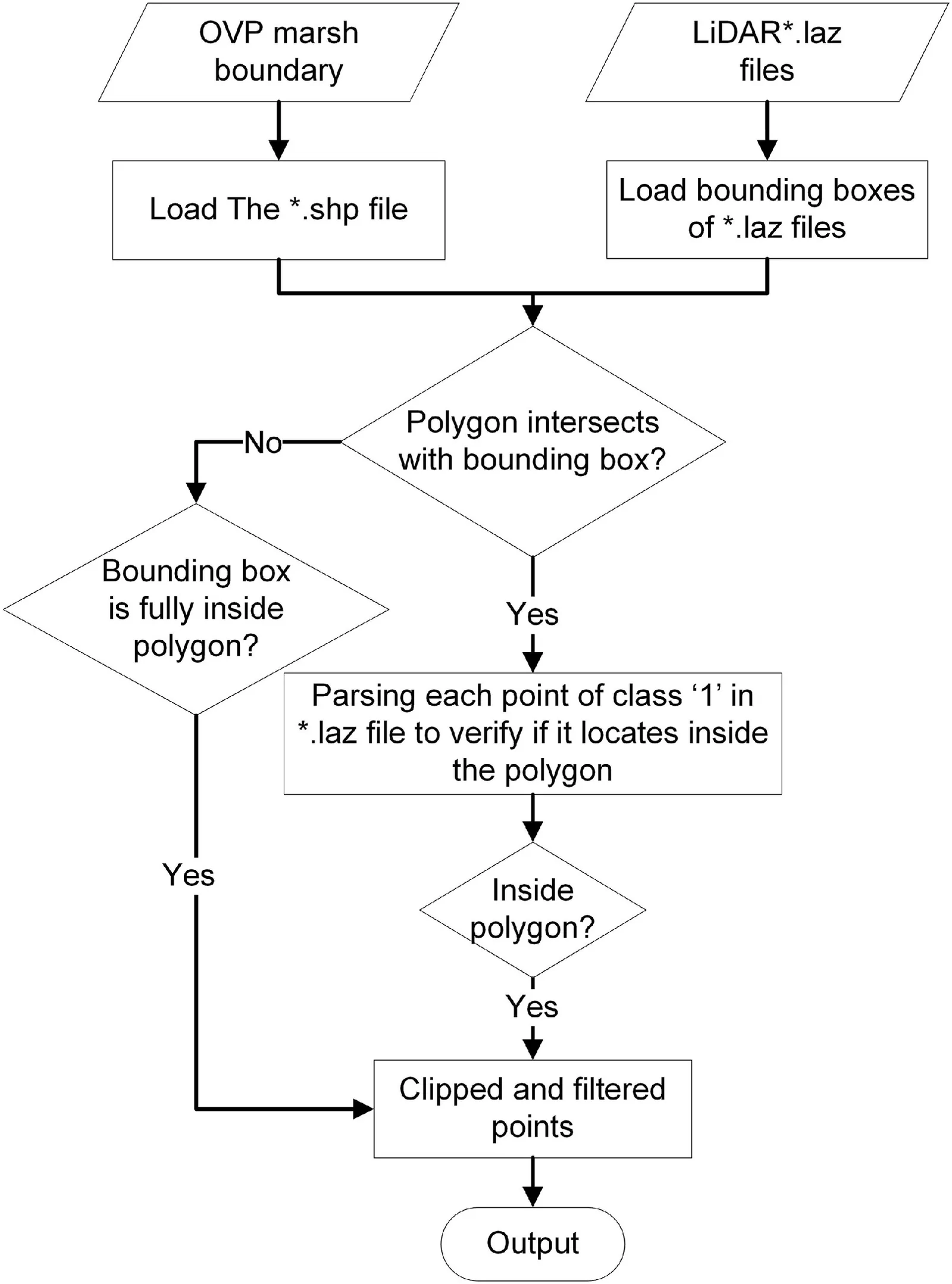
Processing steps for preparing input point clouds from AHN4 airborne laser scanning data. Original LAZ tiles were first intersected with the study area boundary of the Oostvaardersplassen, followed by filtering to retain only points with ASPRS classification ID = 1 (“Unclassified”). The resulting clipped and filtered point clouds serve as input for the tree and shrub extraction workflow.

From the national AHN4 coverage, only tiles intersecting the Oostvaardersplassen nature reserve were selected (Fig. 1). These tiles represented a total of 6,243,044,410 points and a data volume of 42.3 GB. The selected tiles were intersected with a site polygon (ESRI Shapefile) representing the study area boundaries (Fig. 4). These clipped tiles included a total of 490,234,336 points, with a data volume of 3.45 GB. From these clipped tiles, only points pre-classified as ``Unclassified'' (ASPRS classification ID = 1) [19] were retained (Fig. 4). Within the AHN4 dataset, dedicated ASPRS classes are assigned to features such as ground, water, and buildings, whereas vegetation points in the marsh and reedbed areas of the Oostvaardersplassen are predominantly retained in the “Unclassified” category. These points were therefore used as candidate vegetation points for the extraction workflow. This included a total of 132,175,987 clipped and filtered points, with a data volume of 1.08 GB.

### 4.2 Processing workflow for tree and shrub extraction

The extraction and individualization workflow is publicly available at: https://github.com/Jinhu-Wang/Tree_Classification_and_Individualization_In_Marsh_Area.

The implementation is written in C++ and organized into:

• trees3D/ – C++ source code for vegetation classification and tree individualization
• cmake/ – build configuration and environment settings
• resources/ – test data and validation datasets (manually created ground truth)

The workflow consists of two main stages:

### 4.3 Segmentation of tree and shrub points

Input: clipped and filtered point clouds (LAZ).

**Fig. 5:**
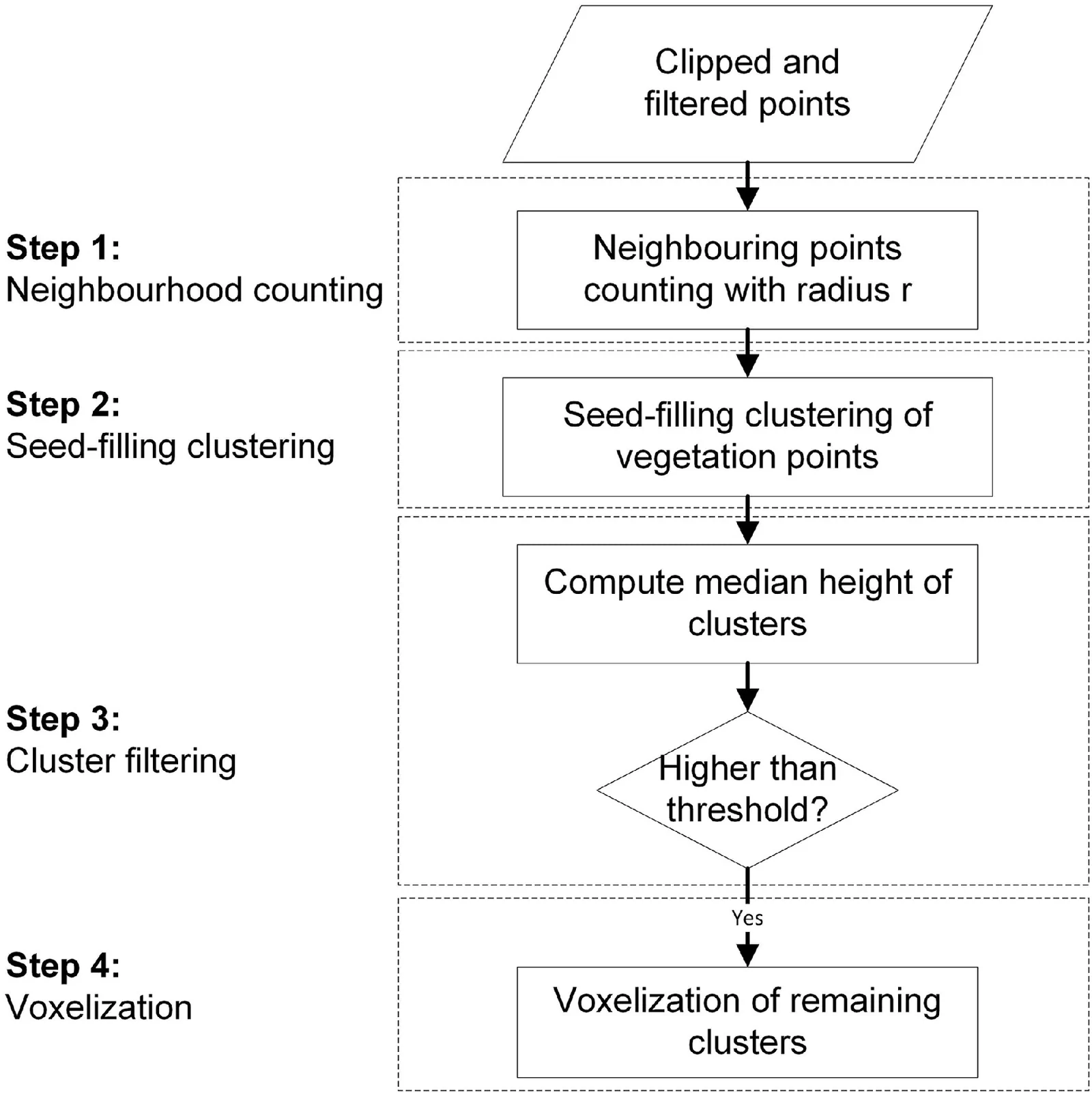
Workflow for segmentation of tree and shrub points from clipped and filtered ALS point clouds. The input points are processed through neighbourhood counting, seed-filling clustering, cluster filtering, and voxelization. The resulting output is a point cloud of segmented tree and shrub points (LAZ format).

Processing steps (see overview in Fig. 5):

1. Neighbourhood counting: For each vegetation point, neighbouring points within a specified radius are counted.
2. Seed-filling clustering: Spatially adjacent points are grouped into clusters using a seed-filling algorithm.
3. Cluster filtering: Median height per cluster is computed. Clusters representing low vegetation are filtered out based on a height threshold, which was set here to −2 m (elevation relative to sea level).
4. Voxelization: Remaining points are divided into 3D voxels with a resolution of 1 m in all three axes. Adjacent voxels are clustered based on spatial proximity.

The parameters used for segmentation were neighbour radius, minimum number of points per cluster, and height threshold (Table 3). These parameter values were selected to represent the spatial structure and connectivity of woody vegetation within marsh and reedbed environments. The output of this stage is a point cloud containing segmented tree and shrub points (LAZ format).

**Table 3:** Parameters used for woody vegetation segmentation and individual tree delineation, including their values and functional interpretation within the marsh and reedbed environments of the Oostvaardersplassen study area.

Task	Parameter	Value	Interpretation
Segmentation	neighbour_radius	1.2 m	Radius used to quantify local point density and identify connected woody vegetation structures
	min_pts_per_cluster	4	Minimum number of neighbouring points required to define a woody vegetation cluster
	height_threshold	−2 m	Threshold used to remove incorrectly classified terrain points and low vegetation clusters
Individual tree delineation	search_radius	1.3 m	Radius used to identify and connect neighbouring voxel components belonging to the same woody element
	voxel_size	1 m	Voxel resolution used for resampling woody vegetation points during individualization
	min_pts_for_seeds	10	Radius used to quantify local point density and identify connected woody vegetation structures

### 4.4 Individual tree delineation

Input: segmented tree and shrub point clouds (LAZ format), consisting of 8,612,922 total points.

**Fig. 6:**
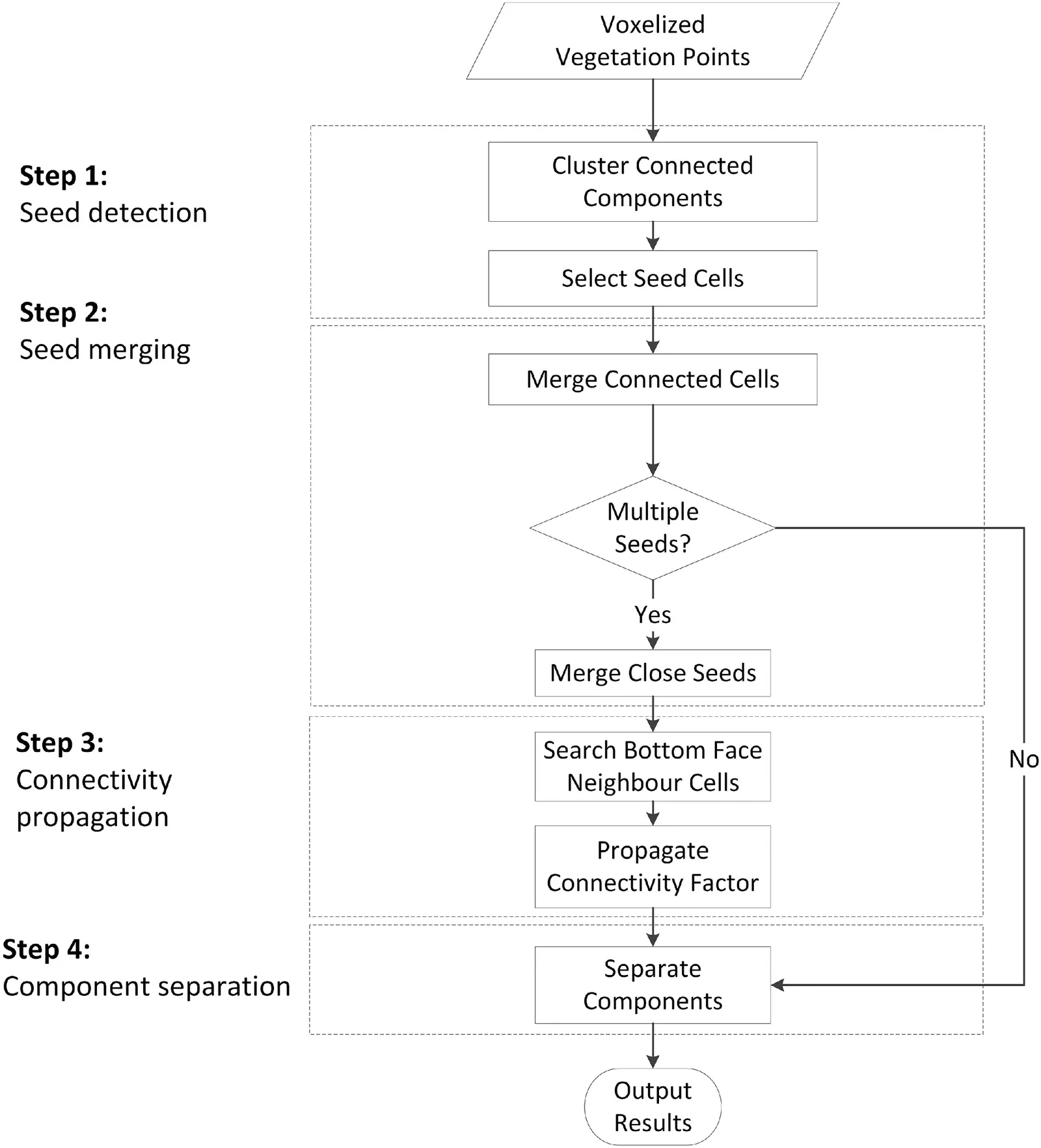
Workflow for individual tree delineation from segmented and voxelized vegetation points. The input point cloud is processed through seed detection (top-down identification of seed clusters), seed merging, connectivity propagation between voxel layers, and component separation. The output consists of point clusters representing individualized trees (LAZ format).

Processing steps (see overview in Fig. 6):

1. Seed detection: Seed cell clusters are identified from upper voxel layers downward.
2. Seed merging: Closely located seed clusters are merged if they represent the same structure.
3. Connectivity propagation: Connectivity coefficients are propagated vertically by inheriting labels from top-face neighbouring voxel clusters.
4. Component separation: Connected voxel components are separated to generate individual tree clusters.

The parameters used for tree individualization were search radius, voxel size, and minimum number of points per seed (details in Table 3). The output consists of point clusters representing individualized trees (LAZ format). Parameter values for individual tree delineation were selected to balance connectivity between neighbouring woody elements while minimizing merging of adjacent tree crowns in densely vegetated patches.

### 4.5 Accuracy assessment

Accuracy assessment was conducted using manually created ground truth datasets. Validation was performed separately for the segmentation of woody vegetation and the individualization of trees and shrubs.

### 4.6 Segmentation validation

**Fig. 7:**
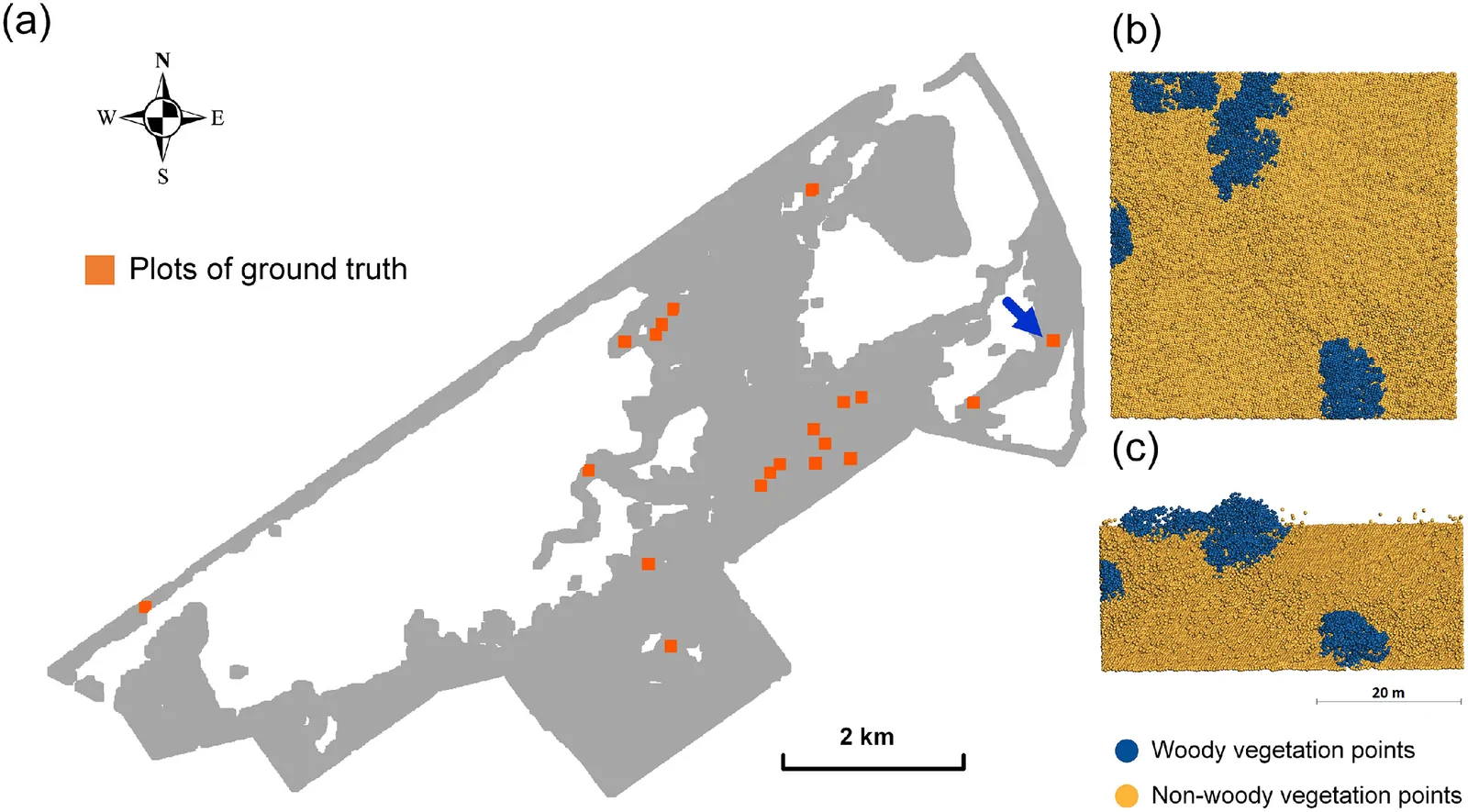
Location and example of manually created ground truth datasets for segmentation validation. (a) Spatial distribution of 20 ground truth plots (50 m × 50 m) within the marsh area containing woody vegetation. (b, c) Top and side views of the plot denoted by the blue arrow in (a), showing manually annotated woody (tree and shrub) and non-woody vegetation points.

A total of 20 square plots (50 m × 50 m) were selected across the study area (Fig. 7), comprising 613,640 points. Trees and shrubs were manually labelled by inspecting the AHN4 point clouds in combination with 8 cm resolution aerial imagery (https://app.pdok.nl/viewer/#x=160000.00&y=455000.00&z=3.0000&background=BRT-A%20standaard&layers=a301ddc7-c26f-42d8-b367-509ae5ae47d0;Actueel_orthoHR;_;1), with each point assigned to one of two classes: “tree” or “non-tree”.

Accuracy (Acc) was computed using a confusion matrix:

$$Acc=\;\frac{TP+TN}{TP+TN+FP+FN}$$

where TP (true positives) are correctly segmented tree points, FP (false positives) are non-tree points incorrectly classified as tree, FN (false negatives) are tree points incorrectly classified as non-tree, and TN (true negatives) are correctly segmented non-tree points.

Point-wise accuracy was calculated for each plot separately, and the overall accuracy for segmentation across all 20 plots was Acc = 0.965 ± 0.028 (mean ± SD).

### 4.7 Individualization validation

**Fig. 8:**
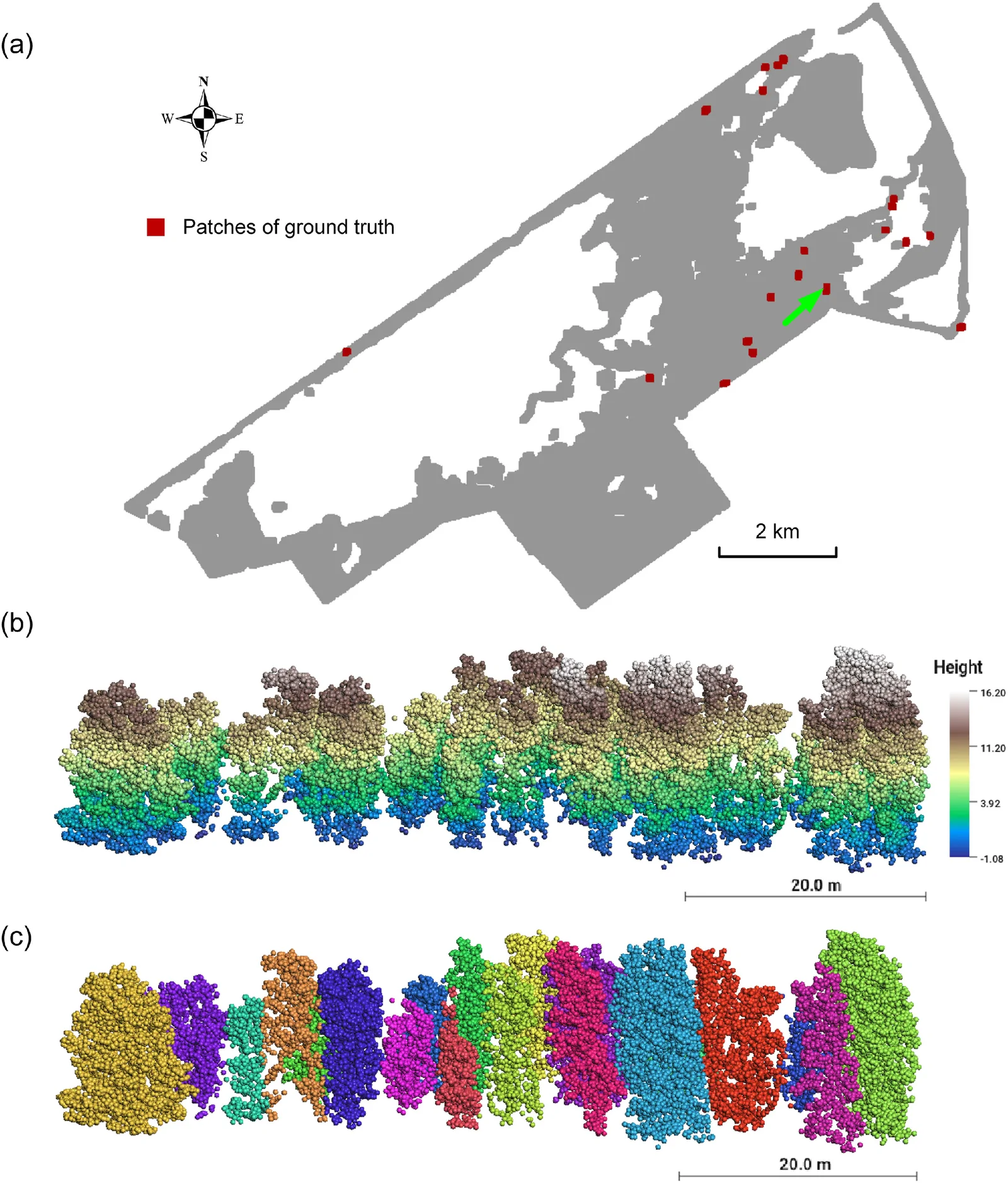
Location and example of manually created ground truth datasets for tree and shrub individualization. (a) Spatial distribution of selected woody vegetation patches used for validation. (b) The patch denoted with the green arrow in (a) contains 21 individuals of varying size and shape, colourized by height. (c) Corresponding manually annotated individual trees and shrubs of the example patch, with distinct colours representing separate individuals.

**Fig. 9:**
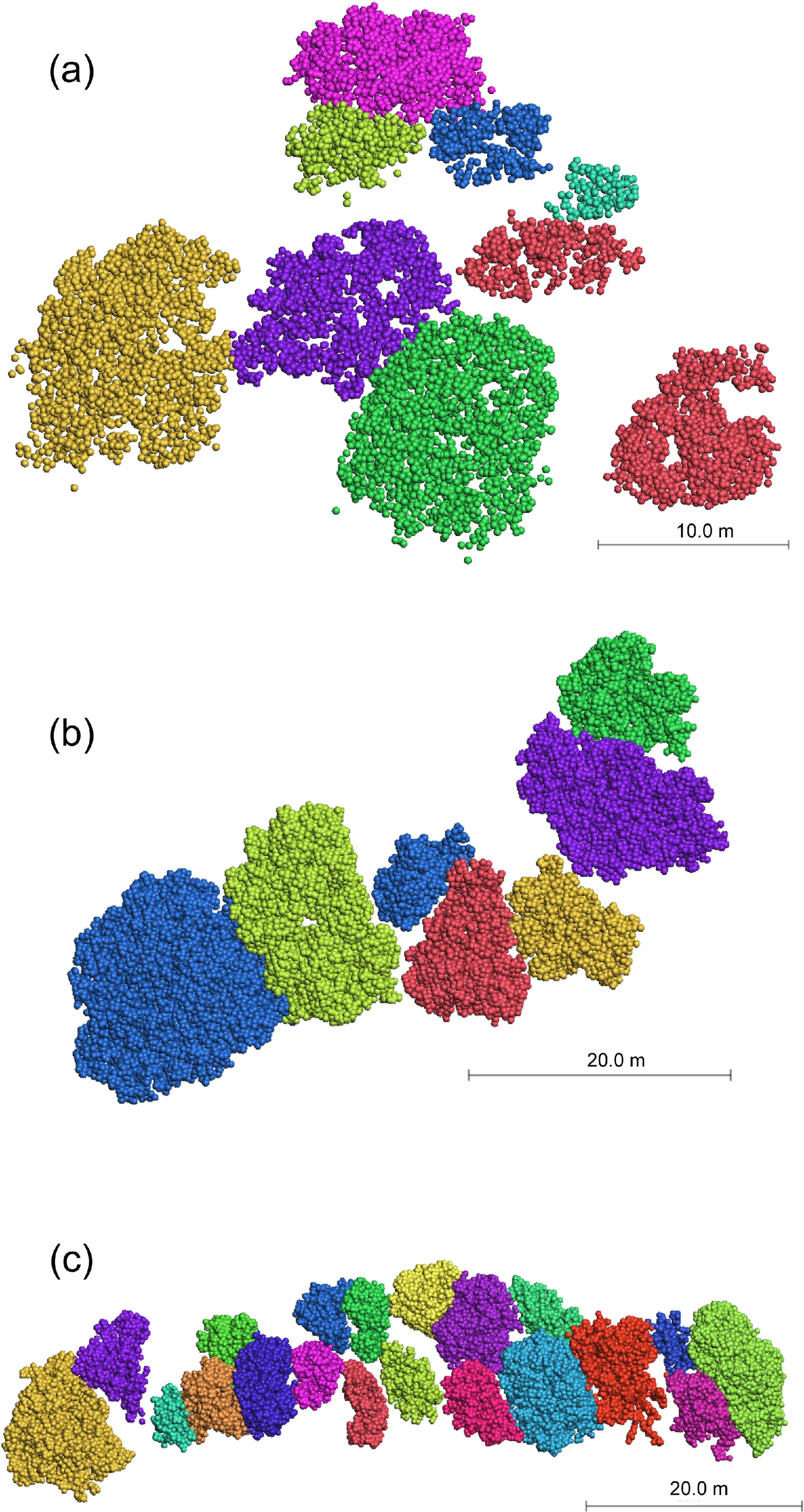
Examples of woody vegetation patches (top view) categorized into three difficulty levels for evaluating individual tree and shrub delineation. (a) Easy patch, with largely isolated trees and shrubs of similar canopy size; (b) medium patch, showing moderate variation in canopy size and spacing; and (c) hard patch, where trees and shrubs are densely packed with highly variable canopy sizes.

A total of 20 tree patches were selected across the study area (Fig. 8) and manually labelled at the individual tree/shrub level, comprising 276,693 points and 214 individual trees and shrubs. Validation data were generated by inspecting AHN4 point clouds in combination with 8 cm resolution aerial imagery. The patches were categorized into three difficulty levels reflecting the complexity of separating individual woody elements (Fig. 9): easy (n = 4), where trees and shrubs are largely isolated and have similar canopy sizes (53 individuals); medium (n = 10), characterized by moderate variation in canopy size and spacing (99 individuals); and hard (n = 6), where trees and shrubs are densely packed with highly variable canopy sizes (62 individuals).

**Fig. 10:**
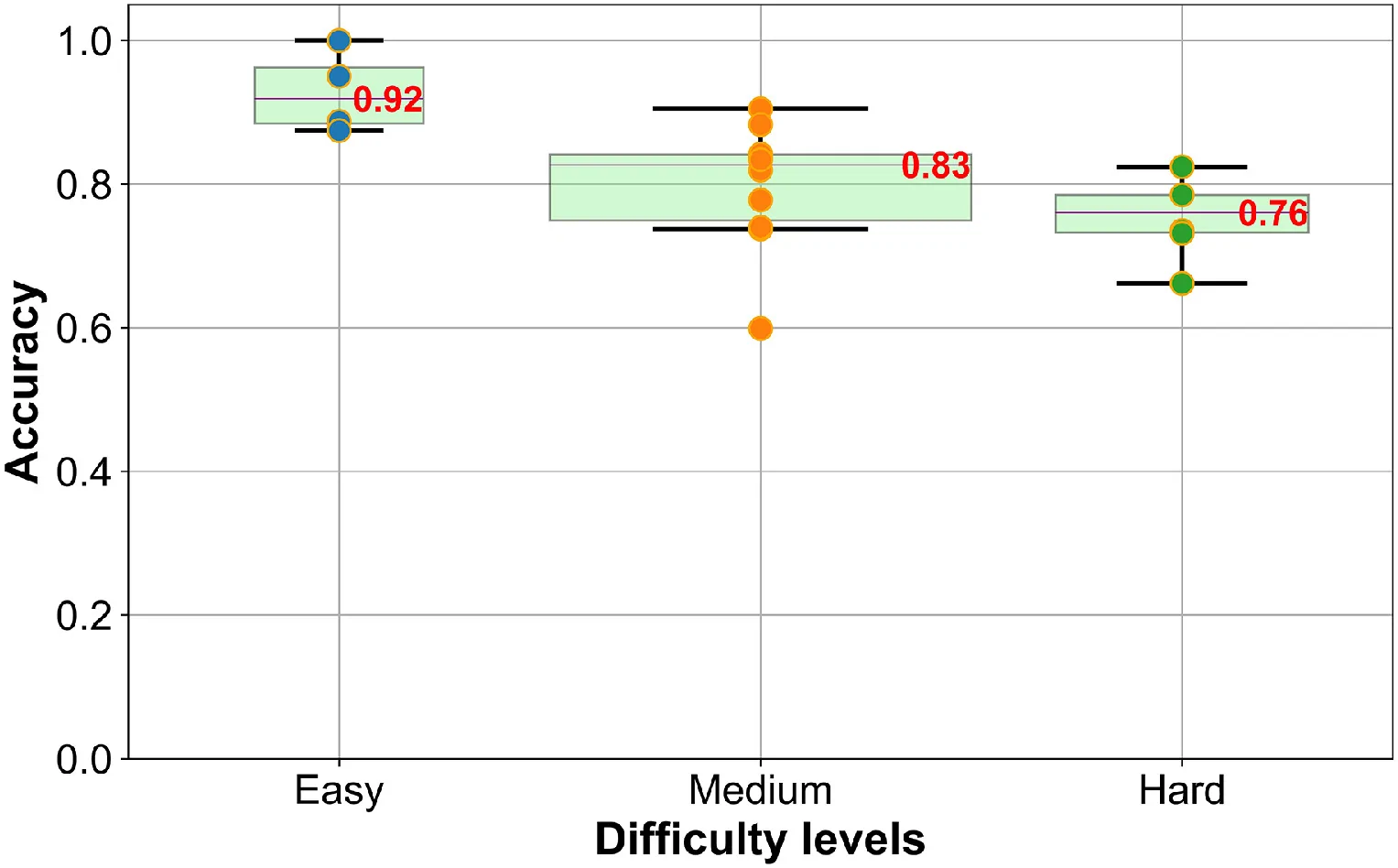
Boxplots of accuracy for tree and shrub individualization across three difficulty levels (easy, medium, hard). Coloured points represent accuracies for individual patches (easy: n = 4; medium: n = 10; hard: n = 6), with median values indicated in red. Difficulty classes correspond to the examples shown in Fig. 9.

Confusion matrices were computed for each patch, and accuracy was calculated using the same formulation as described above. The overall mean accuracy for tree and shrub individualization across all 20 patches was Acc = 0.81 ± 0.096. For the three difficulty levels, mean accuracies were Acc_easy = 0.93 ± 0.058, Acc_medium = 0.80 ± 0.089, and Acc_hard = 0.75 ± 0.057 (see Fig. 10).

**Fig. 11:**
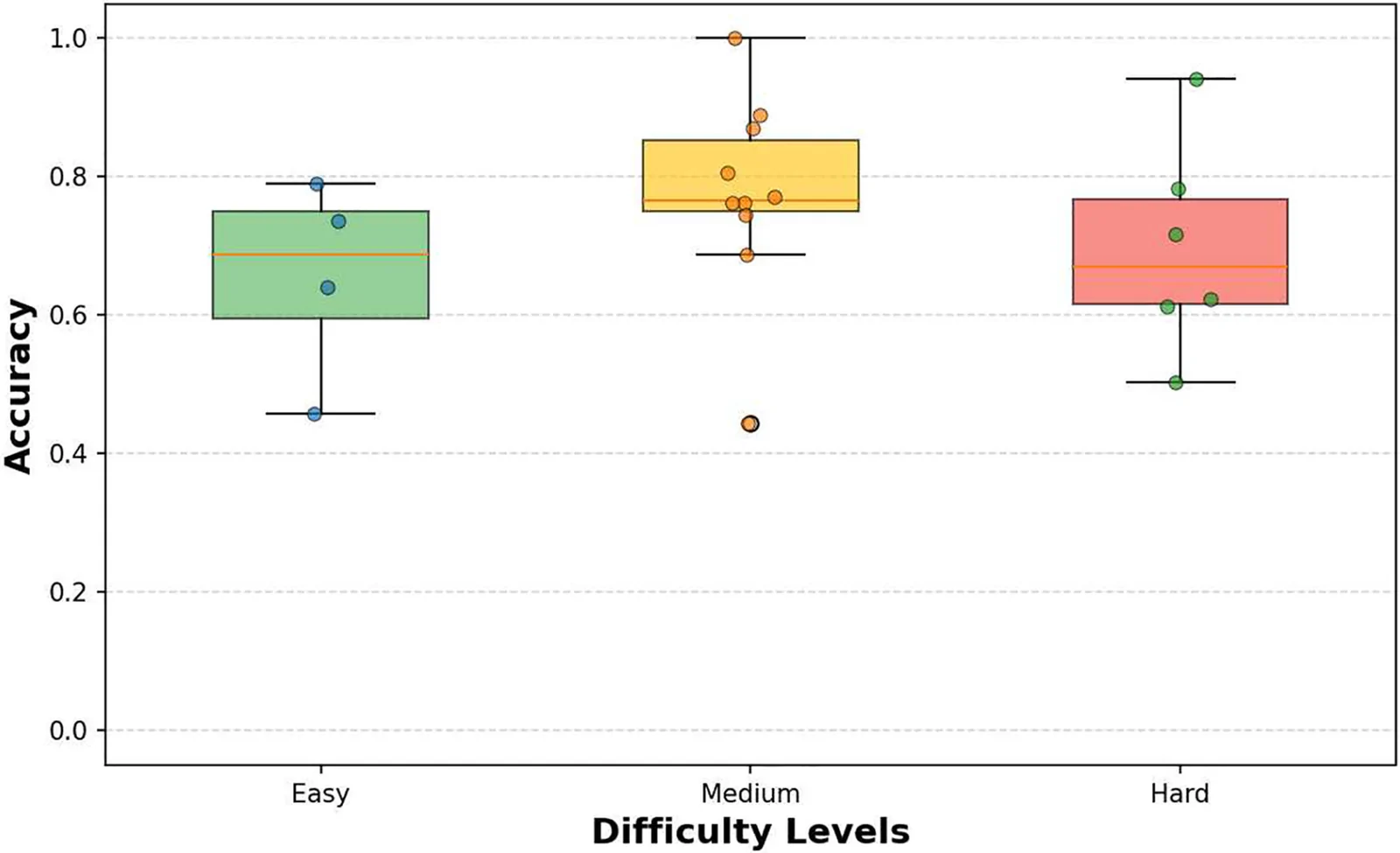
Boxplots of TreeISO benchmark accuracy for individual tree and shrub segmentation across three difficulty levels (easy, medium, hard). TreeISO was implemented in CloudCompare using default parameter settings. Coloured points represent accuracies for individual validation patches (easy: n = 4; medium: n = 10; hard: n = 6).

To provide a reproducible benchmark for the individualization validation datasets, point-level segmentation accuracy was additionally evaluated using the TreeISO plugin implemented in CloudCompare software under default parameter settings. TreeISO is a widely used open-source method for automated individual tree segmentation from point clouds [20] and was included here as an independent benchmark for comparison with the proposed workflow. The default settings used in this benchmark were: initial segmentation regularization strength (λ₁) = 1.0, nearest neighbours (K₁) = 5, and decimated point resolution = 0.05 m; intermediate segmentation regularization strength (λ₂) = 20, nearest neighbours (K₂) = 20, maximum point gap = 2.0 m, and decimated point resolution = 0.1 m; and final segmentation parameters consisting of a relative height-to-length ratio (ρ) = 0.5 and vertical overlap weight = 0.5. The overall mean accuracy across all 20 patches was Acc = 0.73 ± 0.145 (Fig. 11). Across the three difficulty levels, accuracies were Acc_easy = 0.66 ± 0.126, Acc_medium = 0.77 ± 0.139, and Acc_hard = 0.70 ± 0.145.

**Fig. 12:**
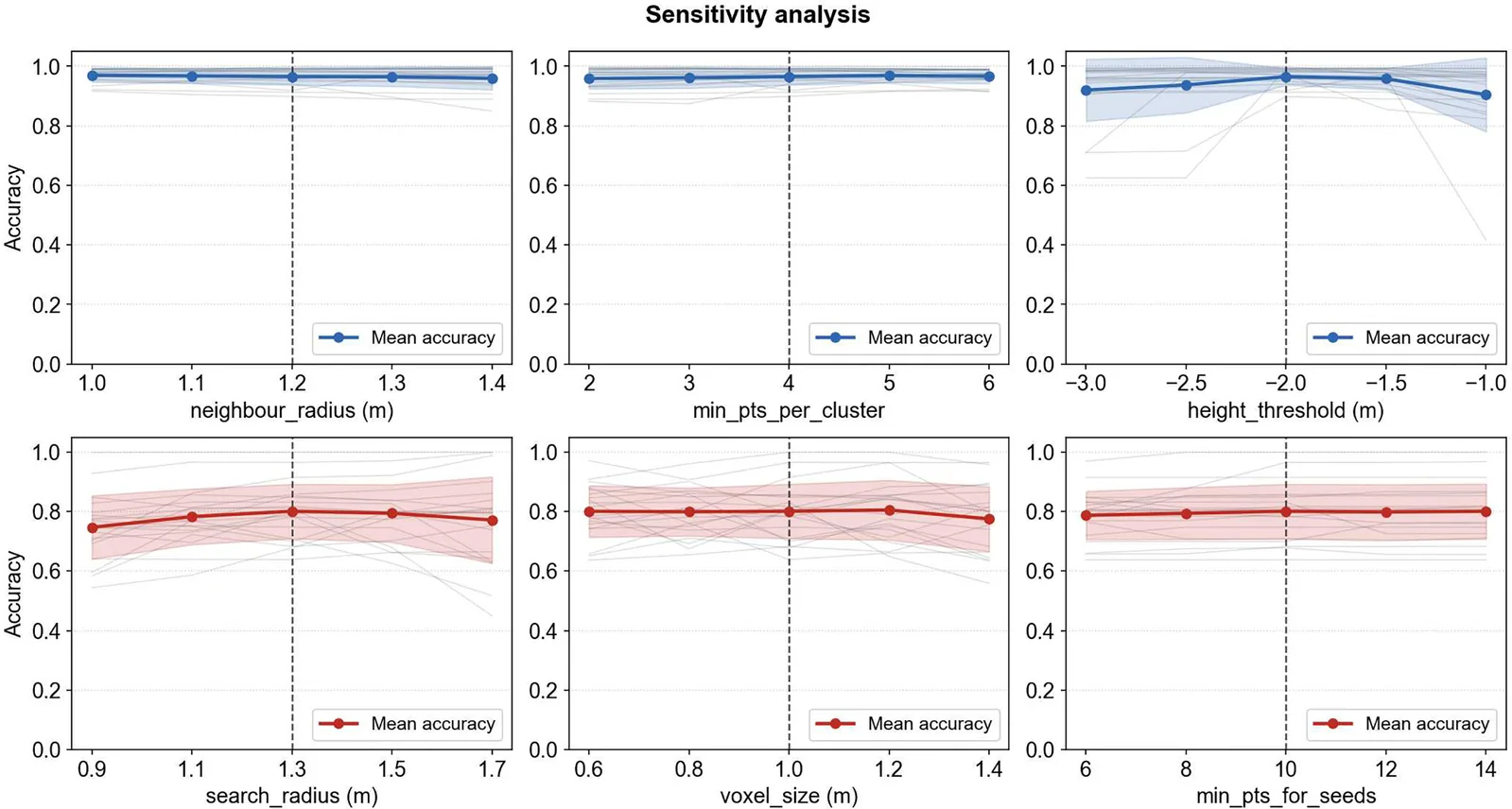
Sensitivity analysis of six workflow parameters used for tree and shrub segmentation and individualization in the marsh area. The top row shows parameters associated with the segmentation stage, whereas the bottom row shows parameters associated with the individualization stage. Dashed vertical lines indicate the default parameter values used in the workflow. Bold lines with circular markers represent mean accuracy across all 20 validation patches, thin grey lines represent accuracies for individual patches, and shaded regions indicate ± one standard deviation. All subplots share the same vertical axis range to facilitate comparison among parameters.

To assess the robustness of the proposed workflow to parameter selection, a sensitivity analysis was performed for the six key parameters listed in Table 3 (Fig. 12). For the segmentation stage (top row in Fig. 12), the tested parameters were neighbour_radius (1.0–1.4 m; default = 1.2 m), min_pts_per_cluster (2–6; default = 4), and height_threshold (−3.0 to −1.0 m; default = −2.0 m). Segmentation accuracies remained relatively stable across the tested parameter ranges, indicating low sensitivity to moderate parameter variation.

For the individualization stage (bottom row in Fig. 12), the tested parameters were search_radius (0.9–1.7 m; default = 1.3 m), voxel_size (0.6–1.4 m; default = 1.0 m), and min_pts_for_seeds (6–14; default = 10). Mean individualization accuracy also remained comparatively stable across the tested ranges. Overall, the sensitivity analysis indicates that the workflow is robust to moderate perturbations in parameter settings and that the selected default values generalize consistently across validation patches.

### 4.8 Post-processing

Post-processing was implemented in Python, with scripts provided in the Zenodo repository (folder 3_Scripts). The workflow consists of two parts: rasterization and boundary extraction.

### 4.9 Rasterization

**Fig. 13:**
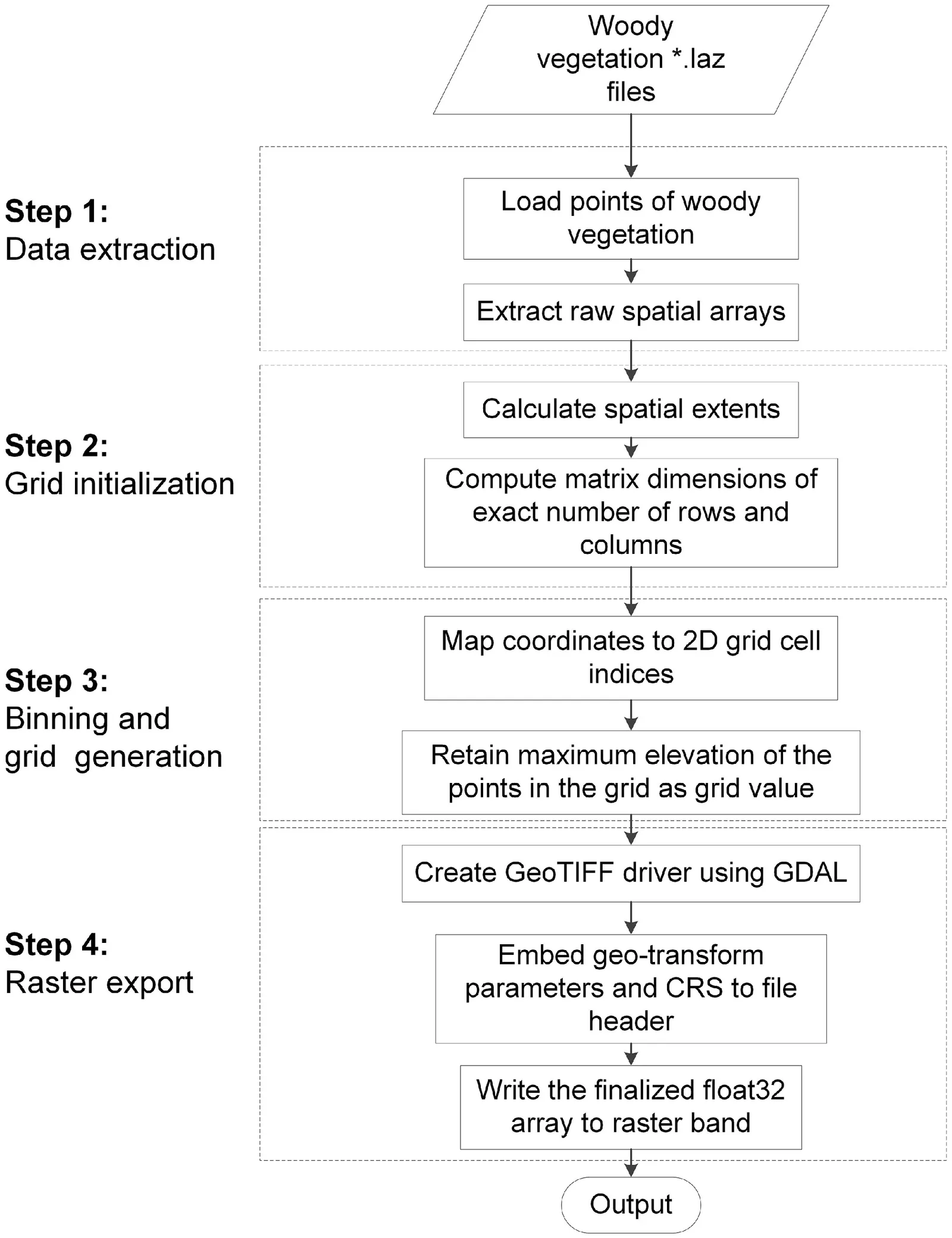
Workflow for rasterization of woody vegetation point clouds into GeoTIFF format, including data extraction, grid initialization, point-to-cell assignment (binning) and grid generation, and raster export.

Rasterization converts extracted woody vegetation point clouds from LAZ format to GeoTIFF raster format using the script `1_generate_tiff.py' (Fig. 13). The procedure includes data extraction, grid definition, point-to-cell assignment, and raster export. Parallel batch processing was implemented using ProcessPoolExecutor to utilize available CPU cores. The output consists of GeoTIFF raster files at 1 m spatial resolution.

The rasterization workflow uses the following Python libraries: *laspy, NumPy, GDAL* (osgeo.gdal, osgeo.osr), and *concurrent.futures*. Processing steps include: (1) reading LAZ files with *laspy*; (2) extracting X, Y, and Z coordinates; (3) defining the raster extent from the point cloud bounding box; (4) computing raster dimensions based on a specified resolution (1.50 m); (5) assigning points to grid cells using index transformation; (6) applying a maximum Z-value per cell using a digital surface model (DSM) approach; (7) creating the GeoTIFF using GDAL; (8) setting geotransform parameters and projection (EPSG:28,992); and (9) writing the raster as float32 with NaN as the NoData value.

### 4.10 Boundary extraction

**Fig. 14:**
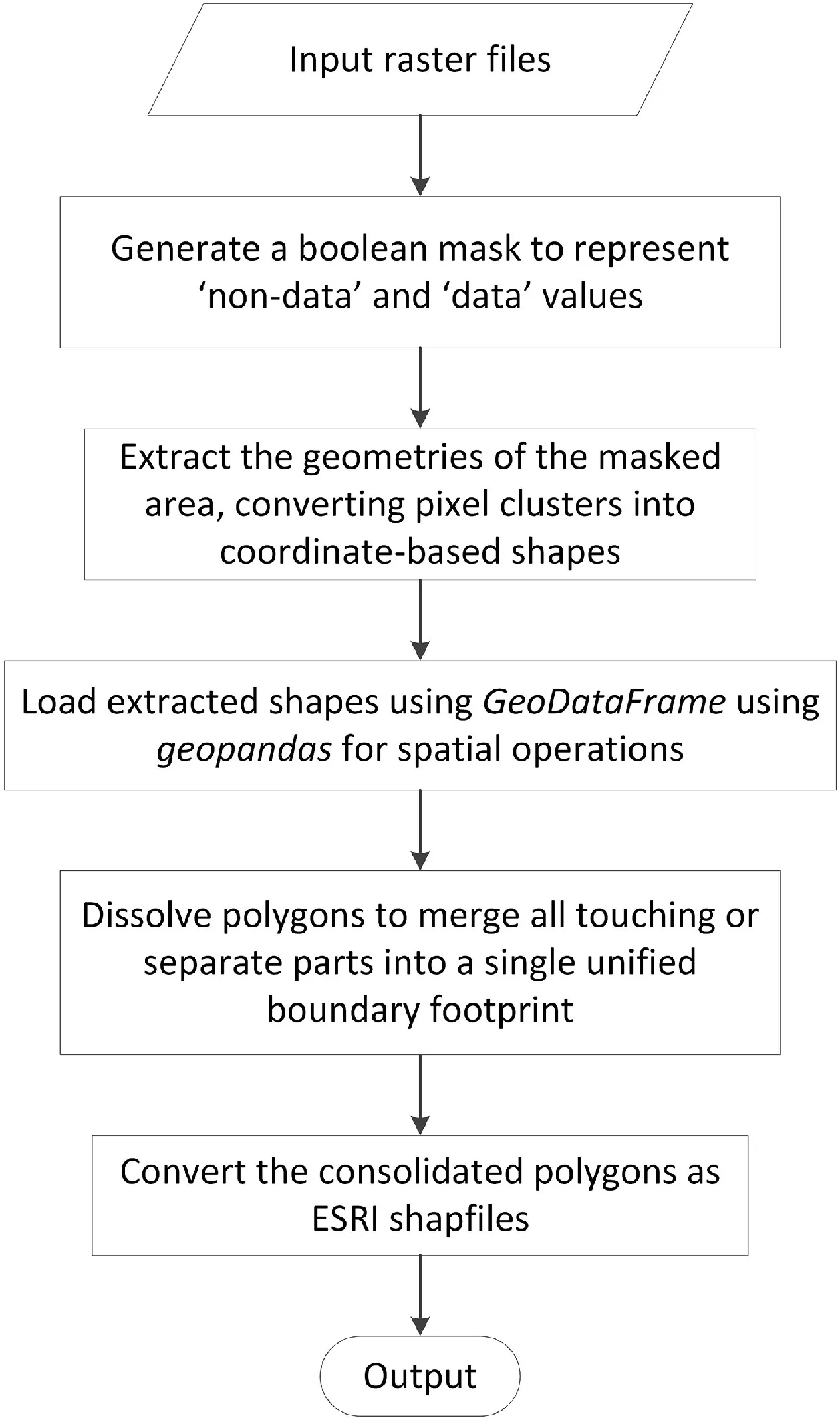
Workflow for boundary extraction from raster masks to vector polygons. Input GeoTIFF rasters are processed by separating NoData and valid data values, extracting shapes from raster cells, dissolving polygons into a single boundary, and exporting the result as ESRI Shapefiles.

Boundary extraction converts raster outputs into vector representations of woody vegetation using the script ‘2_generate_boundary.py’ (Fig. 14). The procedure takes GeoTIFF rasters as input, distinguishes valid data from NoData values, extracts polygonal shapes from raster cells, and dissolves these into a single boundary per raster. Parallel processing was implemented to handle multiple raster files efficiently. The output consists of ESRI Shapefiles representing the spatial footprint of woody vegetation.

The workflow uses the following Python libraries: *rasterio, geopandas, shapely, NumPy*, and *concurrent.futures*. Processing steps include: (1) opening the GeoTIFF raster using *rasterio*; (2) generating a mask to identify valid (non-NoData) cells; (3) extracting shapes from the mask using rasterio.features.shapes; (4) converting the extracted shapes into a GeoDataFrame; (5) dissolving polygons to obtain a single boundary footprint; and (6) exporting the result as an ESRI Shapefile.

### 4.11 Software environment

The workflow uses:

• C++ (tree segmentation and individualization)
• CMake (build configuration)
• Python 3.x (post-processing)
• laspy
• GDAL
• rasterio
• geopandas
• shapely
• NumPy
• CloudCompare (visualization and manual labelling)
• ArcGIS Pro (visualization and inspection)

All scripts, source code, and validation data are publicly accessible via the GitHub repository (https://github.com/Jinhu-Wang/Tree_Classification_and_Individualization_In_Marsh_Area) and Zenodo archive [1].

## Limitations

The dataset is derived from airborne laser scanning data (AHN4) acquired between 2020 and 2022. Spatial resolution, point density, and acquisition conditions (e.g., flight altitude, scan angle, and seasonal timing) are defined by the national survey specifications and may differ when applying the workflow to other ALS datasets, potentially affecting the level of structural detail represented in the point clouds.

The extraction workflow was developed and parameterized for marsh and reedbed environments in the Oostvaardersplassen. Parameter settings (e.g., neighbourhood radius, voxel size, and minimum number of points per cluster) were applied uniformly across the study area and validation plots. Different vegetation structures, canopy densities, or LiDAR acquisition characteristics may require adjustment of these parameters when applied to other environments or datasets.

Ground truth data for accuracy assessment were manually created for a limited number of plots and patches. These validation datasets represent selected areas and predefined difficulty levels and do not provide exhaustive coverage of all vegetation conditions across the study area.

The dataset represents a single wetland system and a specific acquisition period. Changes in vegetation structure occurring after the AHN4 survey are not captured in the dataset.

## Ethics Statement

The authors confirm that they have read and comply with the ethical requirements for publication in Data in Brief and Elsevier’s policies on research ethics and publishing.

The work described in this article does not involve human subjects, animal experiments, or any data collected from social media platforms. The dataset is derived exclusively from publicly available airborne laser scanning data acquired within the Dutch national elevation mapping programme (AHN4) and does not contain personal, sensitive, or ethically restricted information.

## CRediT Author Statement

**Jinhu Wang**: Conceptualization, Methodology, Software, Validation, Formal analysis, Investigation, Resources, Data Curation, Writing - Review & Editing, Visualization; **W. Daniel Kissling**: Conceptualization, Writing - Original Draft, Writing - Review & Editing, Supervision, Project administration, Funding acquisition.

## Declaration of Generative AI and AI-assisted Technologies in the Manuscript Preparation Process

During the preparation of this work, the authors used ChatGPT (OpenAI) to assist with drafting and editing sections of the data article, including improving clarity, structure, and consistency of methodological descriptions, figure captions, and metadata-related text in line with journal guidelines. The tool was also used to refine wording and ensure adherence to the Data in Brief template requirements. After using this tool, the authors critically reviewed, revised, and validated all content and take full responsibility for the content of the published article.

## Data Availability

ZenodoExtracted tree and shrub vegetation from airborne laser scanning (AHN4) in the Oostvaardersplassen wetland, the Netherlands (Original data).
